# Removing Pb(II) Ions from Aqueous Solution by a Promising Absorbent of Tannin-Immobilized Cellulose Microspheres

**DOI:** 10.3390/polym11030548

**Published:** 2019-03-22

**Authors:** Ying Pei, Gaoqiang Xu, Xiao Wu, Keyong Tang, Guozhen Wang

**Affiliations:** 1 School of Materials Science and Engineering, Zhengzhou University, Zhengzhou 450001, China; peiying@zzu.edu.cn (Y.P.); yangwen0132@163.com (G.X.); py885201@126.com (X.W.); kytangzzu@126.com (K.T.); 2 School of Food Science and Engineering, Wuhan Polytechnic University, Key Laboratory for Deep Processing of Major Grain and Oil (Wuhan Polytechnic University), Ministry of Education, Wuhan 430023, China

**Keywords:** tannin, cellulose, microspheres, Pb(II) ions, absorption

## Abstract

Tannin/cellulose microspheres (T/C) were successfully prepared via a facile homogeneous reaction in a water/oil (W/O) emulsion for removing Pb(II) ions from aqueous solution. The structure of the microspheres was characterized by scanning electron microscopy (SEM), Fourier-transform infrared (FTIR) spectroscopy, and a zeta potential test. The effects of pH, adsorbent dosage, contact time, and temperature on adsorption ability were investigated. The results showed that T/C microspheres could combine Pb(II)ions via electrostatic attractions and physical adsorption. Adsorption kinetics could be better described by the pseudo-second-order kinetic model. The adsorption behaviors were in agreement with the Langmuir adsorption isotherm model with a fitting correlation coefficient of 0.9992. The maximum adsorption capacity was 23.75 mg/g from the Langmuir isotherm evaluation at 308K with an initial pH of 5. The results suggested that tannin/cellulose microspheres could be a low-cost and effective adsorbent for removing Pb(II) ions from aqueous solution.

## 1. Introduction

In recent decades, water pollution with heavy metal ions attracted great attention due to their long-term risk to ecosystems and living organisms [1,2]. Lead ions were commonly found in effluents generated from the electroplating and metallurgical industries, as well as the paint, battery, and automobile industries [3]. Lead is well known to be a cumulative poison of the ecological cycle and bioconcentration. It can cause damage to the central peripheral nervous, kidney, and immune systems of people. Particularly for children, the inhalation of Pb(II) is more serious than for adults with the same lead content [4]. Therefore, lead was identified as priority pollution by the United States (US) Environmental Protection Agency (EPA). There is an urgent need for the development of environmentally friendly, facile processes for the treatment of waste waters contaminated with heavy metals.

The adsorption technique is considered to be a low-cost, conventional, and effective method [5]. Considerable research explored the use of biomass-based adsorbent materials such as bark or wood chips for removing soluble heavy metal species from aqueous solution [6]. Tannins are inexpensive and ubiquitous natural polyphenolics with a molecular weight between 500 and 3000 Da; they are extracted from barks, fruits, leaves, and seeds of various plants [7]. Owing to the high content of adjacent phenolic hydroxyls, tannins exhibit excellent chelating affinity toward various metal ions in water [8], as well as showing good adsorbent capacity for cationic dye removal [9]. However, tannins themselves cannot be an ideal adsorbent due to their extremely high solubility in water, which limits their practical application for water treatment. Therefore, great efforts were made to immobilize or chemically modify tannins onto water-insoluble matrices or carriers, including silica beads, hydrotalcite, resin, rayon fiber, zirconium oxide, collagen fibers, agarose, etc. [10].

Cellulose, a biodegradable polysaccharide, is the most abundant biopolymer in plants [11]. It has safe characteristics such as no taste, insolubility, and stability in water and most organic solvents. Due to the multiple hydroxyl groups in its molecular chain, cellulose can be an ideal carrier for tannin immobilization via epichlorohydrin activation [12,13]. Tannin-immobilized cellulose microspheres or hydrogels were successfully fabricated via a facile homogeneous reaction for the removal of cationic dye from aqueous solution in our previous work [14]. In this work, tannin-immobilized cellulose microspheres were fabricated via a homogeneous reaction in a water/oil (W/O) emulsion. The adsorption isotherms, kinetics, and factors affecting Pb(II)adsorption were investigated to evaluate the adsorption ability of tannin/cellulose (T/C) microspheres.

## 2. Experimental

### 2.1. Materials

Cotton linter pulp with an α-cellulose content of more than 95% was purchased from Silver Hawk Chemical Fire Co., Ltd, Gaomi, China. Its average molecular weight (*M*_w_) was evaluated using an Ubbelohde viscometer in a LiOH/urea aqueous solution at 25℃and was calculated, using the equation [*η*] = 3.72 × 10^−2^*M_w_*^0.77^, to be 1.1×10^5^ g/mol according to Cai’s method [15]. Tannins extracted from the fruits of *Areca catechu* (condensed tannin) were provided by the plant extract factory of Guangxi, China. Pb(NO_3_)_2_ was used to prepare a range of different concentrations of Pb(II) ion solutions. All other reagents were of analytical grade and obtained from commercial sources in China.

### 2.2. Fabricationof Immobilized Tannin/Cellulose(T/C) Microspheres

T/C microspheres were fabricated through a facile homogeneous reaction in a W/O emulsion according to our previous work [14]. Briefly, raw cellulose was rapidly dissolved in an NaOH/urea/H_2_O system at low temperature to obtain a cellulose solution according to Zhang’s method [16]. A certain amount of tannin powder was added into the cellulose solution to obtain a tannin/cellulose (T/C) mixture as a water phase (W). Certain paraffin oils, Span 80 and Tween 80, were mixed (the weight ratio *m*_span 80_:*m*_tween80_ is 2:1) in a reactor as the oil phase (O). Subsequently, the W/O was transferred into the immobilized tannin/cellulose (T/C) microsphere suspension after stirring and epichlorohydrin (ECH) activation at 45 °C. Then, the microspheres were washed with methylene chloride and water. The resultant T/C microspheres were freeze-dried for further characterization and tests. Cellulose microspheres without tannins were fabricated using the above method as control samples.

### 2.3. Characterization

#### 2.3.1. FTIR

The dried microspheres were ground into powder and then vacuum-dried for 24 h before FT-IR tests were carried out on an FT-IR spectrometer (Nicolet PROTEGE 460, Madison, WI, USA) in the wavelength range from 4000–500 cm^−1^.

#### 2.3.2. SEM

The morphology of the microspheres was observed on a scanning electron microscope (SIRONTMP, FEI, Hillsboro, OR, USA) at an accelerating voltage of 20 kV. The microspheres were embedded in a low-viscosity epoxy resin (SPI Supplies) and were cut by a razor blade in liquid nitrogen. The cross-sectioned sample was then coated with Au for SEM observation.

#### 2.3.3. Zeta Potential Test

The zeta potentials were measured with a Malvern Zetasizer Nano ZS. Samples for zeta potential measurements were prepared by adding 4 mg of ground nano-sized sample powder in distilled water at different pH values which were adjusted with diluted HCl (1 mol/L) or NaOH solution (1 mol/L).

### 2.4. Adsorption Experiments

A series of batch experiments were conducted to study the Pb(II) ion adsorption mechanism on the T/C microspheres, as well as the adsorption isotherms and kinetics. Bath adsorption experiments were conducted with microspheres in 100-mL storage bottles on a shaker at the speed of 150 rpm in a water bath at a constant temperature until adsorption equilibrium. The suspension was filtered and the concentration of Pb(II)ions in filtrate was measured using the lead dithi zone (H_2_Dz)-PAR (4-(2-Pyridylazo)resorcinol) spectrophotometric method and was calculated from the absorbance at 530 nm using an ultraviolet (UV) spectrophotometer(TU-1950, PERSEE, Beijing, China) [17,18]. The pH of the solution was adjusted to desired values with 0.1N HNO_3_ and 0.1N NaOH.

The adsorption capacity of T/C microspheres and the Pb(II) removal ratio R (%) could be calculated using the following equations:

(1) $$Q\frac{\mathrm{mg}}{g}\;=\;\frac{(C_{0}-C_{t})V}{m},$$

(2) $$R\left(\%\right)\;=\;\frac{C_{0}-C_{t}}{C_{0}}\times 100\%,$$

where *C*_0_ is the initial Pb(II) ion concentration (mg/L), *C_t_* is the residual Pb(II) ion concentration at time *t* (mg/L), *V* is the solution volume (L), and *m* is the microsphere mass.

## 3. Results and Discussion

### 3.1. FTIR

**Figure 1:**
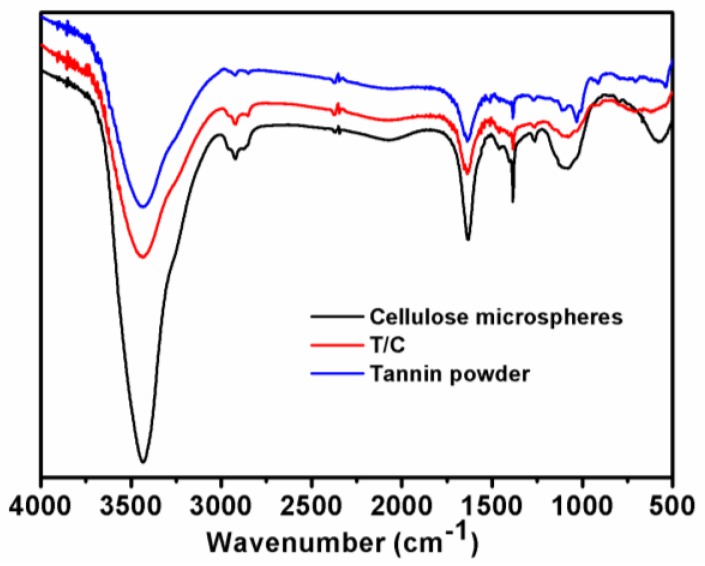
Fourier-transform infrared (FTIR) spectra of tannin powder, cellulose, and immobilized tannin/cellulose (T/C) microspheres.

The FTIR spectra of cellulose microspheres, T/C microspheres, and tannin samples are displayed in Figure 1. The absorption bands around 3400 cm^−1^ were attributed to the hydroxyl stretching vibration bands for cellulose. The intensity in this region decreased and broadened for T/C microspheres, indicating that there were interactions between tannin and cellulose. Cellulose also formed a self-cross-linking structure following the addition of ECH as a cross-linker, resulting in the peak around 1260 cm^−1^, which was associated with C–O stretching of the benzene ring and ether bridges. The wide peaks in the range of 3000–3600 cm^−1^ showed that phenolic groups were intensively present within the nature of tannin. The peaks around 1030 cm^−1^ in the tannin spectrum were due to C–O stretching and CH deformation. The intensity in this region broadened for T/C microspheres, possibly due to the formation of asymmetrical C–O–C bond stretching between tannin and cellulose. Therefore, the above information in the FTIR spectrum gave some evidence of the tannin immobilization on cellulose.

### 3.2. Morphology

**Figure 2:**
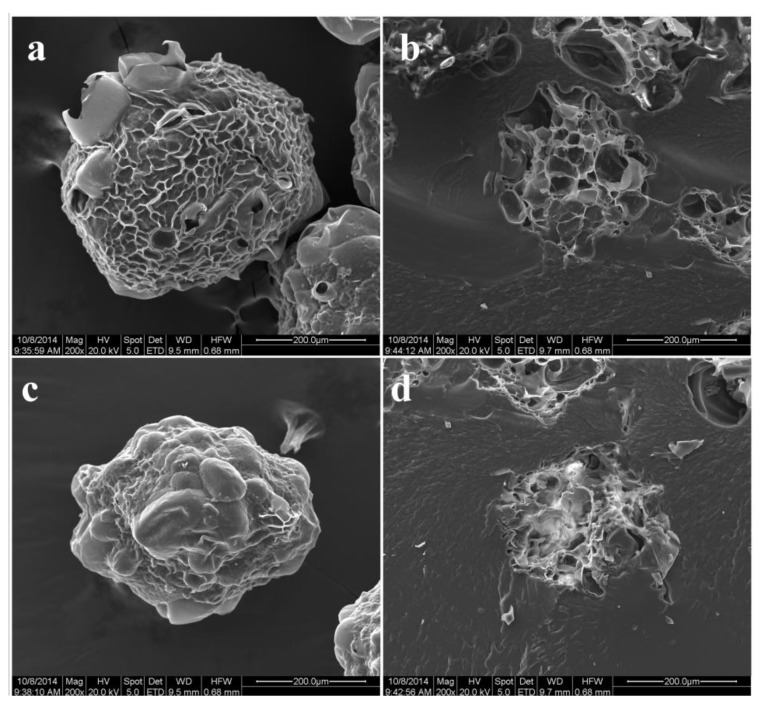
The surface and cross-section morphologies of T/C microspheres (**a**,**b**) and cellulose microspheres (**c**,**d**).

Figure 2 shows SEM images of the T/C microspheres. The microspheres exhibited good spherical shape with mean diameters of 200 ± 15 μm. Both the surface and the interior of microspheres displayed a porous structure which could provide room for heavy metal adsorption. The cross-linking reaction induced the aggregation of cellulose molecules and regeneration from solvent. The porous structure was also seen in the morphology of cellulose microspheres, suggesting that this structure was attributed to cellulose. The aggregation of the rigid cellulose molecules by chemical cross-linking formed the pore walls of cellulose microspheres and provided a support structure for T/C microspheres. Tannins, as small molecules immobilized on cellulose, acted as adsorption sites for heavy metal ions.

### 3.3. Zeta Potentials

The zeta potential of tannin was −22.1 mV when dissolved in neutral water, owing to the ionizations of phenolic hydroxyls in the tannin molecules (Table 1). The zeta potential of cellulose microspheres was −6.46 mV, as a result of the ionization of hydroxyl groups on cellulose d. When the solution pH was in the range of 2–10, the surface charges of the T/C microspheres were all negative due to the immobilized tannin on cellulose. As the pH decreased, the ionization of phenolic hydroxyls was suppressed, especially at lower pH [19]. The negative charge on the surface of T/C microspheres benefited the adsorption of Pb(II) ions in solution.

**Figure 3:**
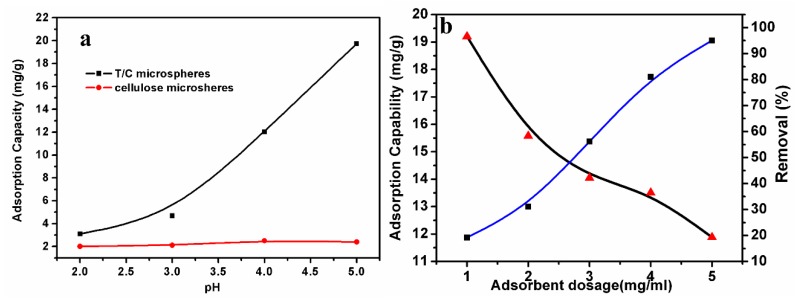
Effect of pH on the removal of Pb(II) ions from the aqueous solution(**a**), and effect of adsorbent dosage on the removal of Pb(II) ions from the aqueous solution (**b**) (*T*/C dosage: 2 mg/mL, Pb(II) ions: 100 mg/L, 308K).

Figure 3a shows the effects of pH on Pb(II) ion adsorption onto the T/C microspheres and cellulose microspheres. Cellulose microspheres exhibited a limited ability of adsorption, suggesting the ineffective combination with Pb(II) ions. After tannin immobilization, T/C microspheres showed a competitive ability for the adsorption of Pb(II) ions. The scheme suggests that a high concentration of OH^−^ favors adsorption, whereas a high concentration of H^+^ ions suppresses the adsorption reaction. An obvious enhancement in adsorption was recorded upon increasing pH in the range of 2–5. The adsorption capacity showed an increase from 3 mg/g to 19 mg/g, demonstrating that the pH of the solution has great influence on lead-ion adsorption onto microspheres. It was reported that solution pH can influence the aqueous chemistry of dye molecules, surface binding sites, and surface charges of the adsorbent [20]. At lower pH, the surfaces of microspheres were occupied by H^+^ protons, leading to a decrease in Pb(II) ion removal capacity. Furthermore, the activity of tannins on cellulose as adsorption sites for removing Pb(II) ions was due to the phenolic hydroxyls on molecules. However, at lower pH, the ionization of phenolic hydroxyls was suppressed, resulting in the lower adsorption capacity of microspheres. With an increase in pH, the increase in negatively charged sites and ionization of tannins led to the increased affinity between microspheres and the positively charged metal ions. This adsorption behavior at pH 2–5 was similar to the cationic dye adsorption on T/C microspheres in our previous work [14].

### 3.4. Effect of Adsorbent Amount

Five microsphere dosages were adopted to evaluate the effect of microsphere amount on Pb(II) ion adsorption, as shown in Figure 3b. The Pb(II) ion removal ratio increased from 12% to 95%, and the Pb(II) ion adsorption capacity onto microspheres decreased from 19 mg/g to 11.5 mg/g upon increasing the microsphere amount. When the proportion was increased from 2 mg/mL to 3 mg/mL, the adsorption capacity of microspheres was generally stable, suggesting the maximum adsorption capacity of microspheres.

### 3.5. Adsorption Isotherms

**Figure 4:**
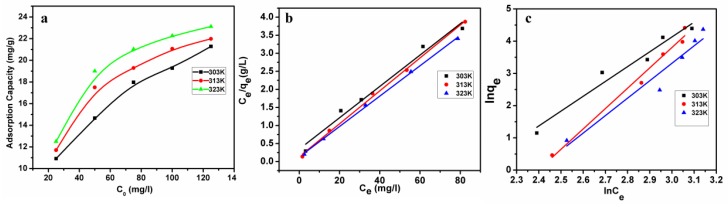
(**a**) Adsorption isotherms of Pb(II) ions at 303K, 313K, and 323K. Conditions: initial pH:2, dosage: 2 mg/mL); (**b**) Langmuir isotherms and (**c**) Freundlich isotherms for Pb(II) ion adsorption onto T/C microspheres at different temperatures.

Adsorption isotherms are very important for designing adsorption systems, revealing the distribution of metal ions between the liquid phase and the solid adsorbentin the equilibrium state during the adsorption process. Figure 4a shows the Pb(II) ion adsorption at 303, 313, and 323K. The adsorption capacity of Pb(II) was significantly increased with the increase of Pb(II) concentration in the low concentration region, and gradually reached an equilibrium at higher Pb(II) concentration. The adsorption capacity of microspheres lightly increased with the increase of temperature, indicating that temperature did not have a significant effect on adsorption capacity. However, adsorption capacity increased obviously from 12.3 to 22.3 mg/g upon increasing the initial concentration of Pb(II) solution. In this work, Langmuir and Freundlich adsorption isotherms were applied for this study to describe the adsorption behaviors of Pb(II) ions onto T/C microspheres, and theyare described in their linear forms according to Equations (3) and (4) [21,22], respectively.

(3) $$\mathrm{Langmuir}:\;\frac{C_{e}}{q_{e}}=\frac{1}{q_{m}K_{L}}+\frac{C_{e}}{q_{m}},$$

(4) $$\mathrm{Freundlich}:\;\ln q_{e}=\ln K_{f}+\frac{\ln C_{e}}{n},$$

where *C_e_* (mg/L) is the Pb(II) equilibrium concentration, *q_m_* (mg/g) is the maximum amount of Pb(II) adsorbed onto microspheres, *K_L_* (L/mg) is a constant related to the heat of adsorption, *K_F_* (L/g) represents the adsorption capacity when *C_e_* is equal to 1, and 1/*n* represents the degree of dependence of adsorption on the equilibrium concentration.

Figure 4b,c show the Langmuir and Freundlich isotherms for Pb(II) concentration, and the according parameters are shown in Table 1. The monolayer capacity *q_m_* was an approximate evaluation of adsorption capacity for Pb(II) adsorption. Figure 4c presents Freundlich isotherms for Pb(II) adsorption. According to a previous report [21], the adsorption is easy when 0.1 < 1/n ≤ 0.5. As shown in Table 2, the 1/n value lay in the range from 0.15 to 0.21 when temperature was varied from 303K to 323K, indicating that the microspheres were extremely suitable for adsorbing Pb(II) ions. The adsorptive behavior of microspheres better followed the Langmuir isotherm due to the high correlation coefficient. Compared to other biomass-based adsorbents for removing Pb(II) from aqueous solution [23], the adsorption capacity of T/C microspheres was competitive.

### 3.6. Adsorption Kinetics

**Figure 5:**
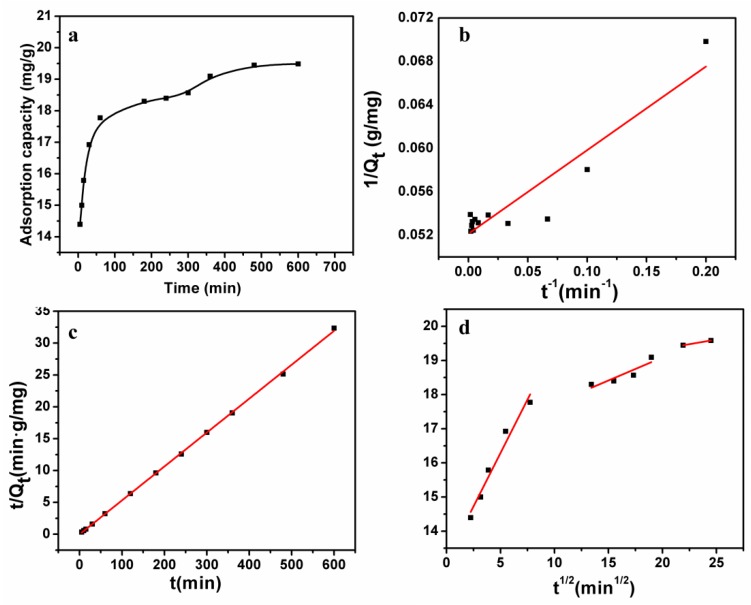
(**a**) Effects of contact time on Pb(II) ion adsorption onto T/C and cellulose microspheres. (**b**) Pseudo-first-order model, (**c**) pseudo-second-order model, and (**d**) intraparticle diffusion model for Pb(II) ion adsorption onto T/C microspheres(initial concentration:100 mg/L, pH:5, *T*:303K).

Figure 5a shows the curves of adsorption capacity of microspheres against contact time. Within 60 min, the adsorption capacity obviously increased, showing the rapid adsorption rate of microspheres. An adsorption equilibrium stage appeared after 480 min without a further increase in adsorption capacity. Figure 5a shows that the adsorption of Pb(II) increased with time from 0 to 300 min, and then became almost constant up to the end of experiment. As presented, the adsorption capacity of Pb(II) significantly increased to 18mg/g in the first 60 min. We reasoned that the mass transfer resistance was greatly reduced due to the porous structure of T/C microspheres, which favors the easy access of Pb(II) to the adsorption site (phenolic group), resulting in a relatively high adsorption rate.

In order to investigate the adsorption process and mechanism for Pb(II) ion adsorption onto T/C microspheres, three kinetic models were implemented including pseudo-first-order, second-order, and intraparticle diffusion models, shown in the equations below [24,25,26,27].

(5) $$\text{The pseudo-first-order}:\frac{1}{Q_{t}}\;=\;\left(\frac{k1}{Q_{1}}\right)\left(\frac{1}{t}\right)+\frac{1}{Q_{1}},$$

(6) $$\text{The pseudo-second-order}:\frac{t}{Q_{t}}\;=\;\frac{1}{k_{2}Q_{2}{}^{2}}+\frac{t}{Q_{2}},$$

(7) $$\text{Intraparticle diffusion}:\;Q_{t}\;=\;k_{p}t^{1/2}+C,$$

where *t* is the adsorption time (min), *Q*_1_ (mg/g) is the maximum adsorption capacity for the pseudo-first-order adsorption, *Q*_2_ (mg/g) is the maximum adsorption capacity for the pseudo-second-order adsorption, *k*_1_ (1/min), *k*_2_ (min·g/mg), and *k_p_* (mg·g^−1^min^1/2^) are the rate constants for the pseudo-fist-order, pseudo-second-order, and intraparticle diffusion models, respectively, and *C* is a constant.

These fitting curves are displayed in Figure 5b,c, and the parameters were calculated from these curves (shown in Table 3). Values of *Q_cal_* (mg/g) from the pseudo-first-order model and pseudo-second-order model were 18.8 mg/g and 19.9 mg/g respectively. From the experimental data of equilibrium, we can determine that the adsorption capacity was 19.5 mg/g, which is close to the value of *Q_cal_*_2_ (mg/g). Furthermore, the correlation coefficient *R*^2^ = 0.9985 shows that the pseudo-second-order model fit the experimental data better than the pseudo-first-order model, suggesting that the pseudo-second-order mechanism is predominant in the adsorption process, and the chemisorptions might have been the rate-limiting step that controlled the adsorption process. Figure 5d shows the multilinear plots of intraparticle diffusion processes of Pb(II) adsorption onto T/C microspheres, indicating that three steps were taken. The first stage was the process where the Pb(II) ions diffused from the solution through the fluid interface membrane onto the surface of the microspheres. The second stage was the intraparticle diffusion of Pb(II) ions from the external surface through the pores of the microspheres. The film diffusion was a rapid process, while the intrapaticle diffusion was a gradual process, resulting in a larger value of *kp1* than *kp2*. The last stage was the final equilibrium, where Pb(II) ions were adsorbed onto tannins residing as active sites on the internal surface of the pores. At that stage, the diffusion was much slower due to the Pb(II) ion concentration in solution and the repulsive forces from the large amounts of Pb(II) ions on the T/C microspheres.

### 3.7. Suggested Pb(II) Ion Removal Mechanism with T/C Microspheres

**Figure 6:**
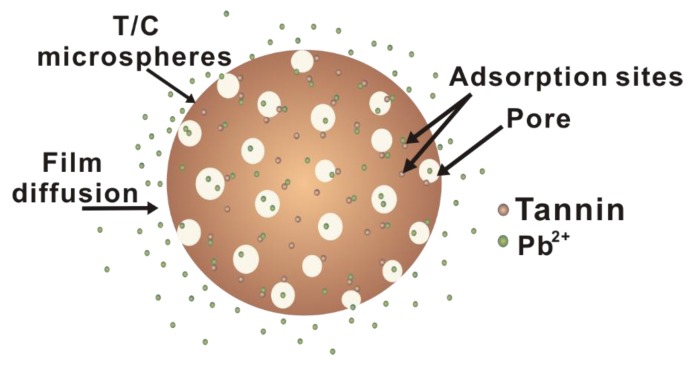
Schematic illustration of the process and mechanism of Pb(II) ion adsorption onto T/C microspheres.

This work presents a possible mechanism for the adsorption of Pb(II) onto T/C microspheres based on a single-factor discussion of adsorption batch experiments and the morphology characterization of microspheres (Figure 6).The pH is a key factor influencing the adsorption of Pb(II) onto microspheres, as the zeta potential analysis showed that the pH of the solution will affect the surface charge of the microspheres. When pH increases the negative charge increases; thus, under the condition of higher pH, T/C microspheres and Pb(II) have a strong combination, which means that the removal effect is good.

At the same time, the composite microspheres had a porous structure composed of cellulose, and the stationary cellulose molecule chain would rather provide adsorption sites for the adsorption of Pb(II). The initial phase of adsorption involved diffusion from the solution to the surface of the composite microspheres driven by heavy metal ions, some of which were adsorbed onto the tannin on the surface of the microspheres, and others which passed through the pores of the microsphere surface to the inner mass of the microspheres. As adsorption continued, the concentration of Pb(II) gradually reached equilibrium, and the adsorption rate subsequently slowed down. With the increase of Pb(II) ions in the microspheres, electrostatic repulsion gradually formed with the Pb(II) ions in solution, which resulted in the decrease in adsorption rate.

## Figures and Tables

**Table 1:** Zeta potentials of the immobilized tannin/cellulose (T/C) microspheres at different pH values.

Sample	pH	Zeta Potentials (mV)
Tannin powder	7	−22.1
T/C microspheres	2	−3.20
	4	−9.90
	6	−12.2
	8	−13.0
	10	−15.8
Cellulose microspheres	7	−6.46

**Table 2:** Parameters for Pb(II) ion adsorption onto T/C microspheres.

Temperature	Langmuir Parameters			Freundlich Parameters		
	*q_max_* (mg/g)	*K_L_* (L/mg)	*R* ^2^	*1/n*	*K_f_* (mg/g)	*R* ^2^
303K	23.0203	0.1260	0.9832	0.2094	8.3955	0.9632
313K	21.9154	0.3397	0.9980	0.1562	11.067	0.9864
323K	23.7586	0.3244	0.9992	0.1746	11.230	0.9452

**Table 3:** Kinetic models and parameters for Pb(II) ion adsorption onto theT/C microspheres.

Model	Parameters	*R* ^2^
Pseudo-first-order	*k*_1_ = 1.8582 min^−1^	0.8371
	*Q_cal1_* = 18.80 mg·g^−1^	
Pseudo-second-order	*k*_2_ = 0.0053 g·mg^−1^·min^−1^	0.9985
	*Q_cal2_* = 19.99 mg·g^−1^	
Intraparticle diffusion	*K_t1_* = 0.6257 mg·g·min^−1/2^	0.9664
	*C*_1_ = 13.16 mg·g^−1^	
	*K_t2_* = 0.0684 mg·g·min^−1/2^	0.9637
	*C*_2_ = 17.36 mg·g^−1^	
	*K_t3_* = 0.0527 mg·g·min^−1/2^	0.9960
	*C*_3_ = 18.29 mg·g^−1^	
